# Conference proceedings from the Western Canadian Neuromuscular Conference (WCNMC) — September 27–29, 2024, Calgary, Canada

**DOI:** 10.1177/22143602251389759

**Published:** 2025-10-21

**Authors:** Gordon Jewett, Collin Luk, Homira Osman, Gerald Pfeffer

**Affiliations:** 1 Department of Clinical Neurosciences, Cumming School of Medicine, 2129 University of Calgary, Calgary, AB, Canada; 2 Hotchkiss Brain Institute, Cumming School of Medicine, 2129 University of Calgary, Calgary, AB, Canada; 3 459728 Muscular Dystrophy Canada, Toronto, Canada; 4 Neuromuscular Disease Network for Canada (NMD4C), Ottawa, Canada; 5 Department of Medical Genetics, Alberta Child Health Research Institute, Cumming School of Medicine, 2129 University of Calgary, Calgary, AB, Canada

**Keywords:** neuromuscular disease, conference, conference proceedings, transdisciplinary

## Abstract

The Western Canadian Neuromuscular Conference (WCNMC) is a conference focused on neuromuscular medicine that was held in Calgary, Alberta from September 27–29, 2024. Although WCNMC has a history going back to 2010 as a regional event, the most recent iteration of the conference aimed to expand this as a national meeting. A collaboration with Muscular Dystrophy Canada and the Neuromuscular Network for Canada (NMD4C) helped to achieve this goal and encourage participation from across Canada. Other goals for this event were to increase the opportunities for trainees, showcase new research, and integrate new topics into the program. The sessions included a clinicopathologic case series for the first time, which was led by neuromuscular fellows from across Canada. Sessions covering topics in neuromuscular disease focused on challenging diagnostic situations, and therapeutic developments for diseases including spinal muscular atrophy, Pompe disease, and TTR amyloidosis. A separate session covered genetic neuromuscular diseases, with a focus on conditions with genetic founder effects in western Canada. Finally, a transdisciplinary session was included, which discussed patient-focused issues in neuromuscular care, as well as preliminary results from the Burden of Inherited Neuromuscular Disease (BIND) study, on the indirect costs of living with a neuromuscular disorder. A research symposium at the conclusion of the event focused on trainee-led research but also included lectures regarding antisense therapies in neuromuscular disease and updates in research on amyotrophic lateral sclerosis.

## Introduction

The 2025 Western Canadian Neuromuscular Conference (WCNMC) was a national conference focused on the clinical investigation and management of neuromuscular disorders (NMD). Founded in 2010, the WCNMC was traditionally run as a regional conference, rotating through host cities of Western Canada (Table 1). The conference returned to an in person format for the first time since the SARS-CoV-2 pandemic in Calgary from September 27–29, 2024. The organizing committee included Dr Gerald Pfeffer (University of Calgary) as conference chair, and Drs Gordon Jewett (University of Calgary) and Collin Luk (University of Calgary) serving as co-chairs. Logistical support was provided by Spark Event Collective (Calgary, Canada).

**Table 1.** Previous Western Canadian Neuromuscular Conferences and host cities.

Year	Host City	Chair
2010	Edmonton, Alberta	Dr Zaeem Siddiqi (University of Alberta)
2012	Banff, Alberta	Dr Sameer Chhibber (University of Calgary)
2015	Vancouver, British Columbia	Dr Kristine Chapman (University of British Columbia)
2017	Edmonton, Alberta	Drs Cecile Phan and Zaeem Siddiqi (University of Alberta)
2021	Virtual*	Drs Kerri Schellenberg (University of Saskatchewan) and Kristine Chapman (University of British Columbia)

*Delayed by the SARS-CoV-2 pandemic

The organizing committee set out four goals that were met in this conference, (1) to transform the WCNMC into a national conference in neuromuscular medicine, (2) to diversify the content in the conference to have broad relevance to neuromuscular specialists and physiatrists, (3) to provide an expanded role for trainees at the conference, (4) to trial the inclusion of a research symposium at the end of the conference.

### The WCNMC as a national conference

The WCNMC was successfully upgraded to a national event due to an early partnership established with Muscular Dystrophy Canada (Dr Homira Osman) and the Neuromuscular Network for Canada (NMD4C, national PI Dr Hanns Lochmüller, University of Ottawa). These partnerships helped to promote the event across Canada, and subsidized attendance for neuromuscular trainees from across Canada. The NMD4C annual meeting was hosted the day prior to the start of the WCNMC, which was reciprocally beneficial by increasing attendance at both meetings.

### Diversifed scientific program

Past WCNMC focused on updates in neuromuscular disease and clinical care, with content mainly directed to neurologists. We broadened the focus to include content of specific interest to physiatry specialists, who play a key role in management of neuromuscular diseases in Canada. We also included a transdisciplinary session (detailed below) to provide new perspectives and topic coverage, including increased focus on patient experiences, education, and the financial burdens associated with NMD.

### Expanded trainee participation

The welcome reception on September 27^th^ included a trainee led interactive clinicopathologic case series. Five challenging cases were presented by residents or neuromuscular fellows from five different training programs (Table 2). This was a highlight of the conference. Trainees were supported by additional satellite social and networking events organized by NMD4C. Trainees also led much of the content in the Research Symposium (described below).

**Table 2.** Presenters and presentation topics at WCNMC 2024 day 1.

Speaker and affiliation	Presentation title
*Clinicopathologic case series*	
Dr Zahra Aleisha, University of Toronto	Clinicopathologic case series presentation
Dr Francis Jalipa, University of Alberta	Clinicopathologic case series presentation
Dr Lola Lessard, University of Ottawa	Clinicopathologic case series presentation
Dr Julia Newton, University of Saskatchewan	Clinicopathologic case series presentation
Dr Tefani Perera, University of Calgary	Clinicopathologic case series presentation
*Topics in Neuromuscular Disease*	
Dr Stephen McNeil, University of Calgary	Diagnostic mimics in the EMG lab
Dr Hans Katzberg, University of Toronto	Myasthenia Gravis
Dr Kristine Chapman, University of British Columbia	Incorporating Patient Reported Outcome Measures (PROMs) and objective outcome measures into clinical practice - a value based health care approach
Dr Zaeem Siddiqi, University of Alberta	Update on autonomic nervous system disorders
Dr Michelle Mezei, University of British Columbia	TTR Amyloidosis
Dr Chris White, University of Calgary	Finding the signal in the noise: EMG in the ICU
Dr Cam-Tu Nguyen, Universite de Montreal	Newborn screening and the evolving care of spinal muscular atrophy
Dr Colleen O’Connell, Dalhousie University	Spinal Muscular Atrophy and bulbar outcomes
*Transdisciplinary session*	
Dr Homira Osman, Muscular Dystrophy Canada	Engagement and support for people living with neuromuscular disease
Dr Ming Chan, University of Alberta	Rehabilitation outcomes in the gene therapy era
Dr Jodi Warman, University of Ottawa	The economic burden of neuromuscular disease, results from the BIND study

### Including a research symposium at WCNMC

We included a research symposium featuring three invited and five best abstract lectures, and 11 posters. Trainee awards were presented for Best Research Presentation and Best Poster Presentation.

The conference was structured around four major sections with several lectures focused on topics of broad interest in neuromuscular disease. A transdisciplinary session was included for the first time. The last day included topics on genetic neuromuscular diseases, with a special focus on those that have founder effects in Western Canada. The research symposium concluded the conference. Below we summarize the conference sessions. The presenters and their presentation titles are listed in Table 2 and Table 3.

**Table 3.** Presenters and presentation topics at WCNMC 2024 day 2.

Speaker and affiliation	Presentation title
*Genetic neuromuscular disease*	
Dr Aneal Khan, MAGIC clinic, Calgary	Pompe disease
Dr Hanns Lochmuller, University of Ottawa	Congenital myasthenic syndromes
Dr Omid Kiamanesh, University of Calgary	Cardiac comorbidities of neuromuscular disease
Dr Cynthia Gagnon, University of Sherbrooke	Update on myotonic dystrophy
Dr Micheil Innes, University of Calgary	Founder neuromuscular diseases in Western Canada
Dr Kerri Schellenberg, University of Saskatchewan	Spinal bulbar muscular atrophy
*Research Symposium*	
Dr Toshifumi Yokota, University of Alberta	Translational research in neuromuscular disease
Dr Minh Dang Nguyen, University of Calgary	Of Gut, Brain & Sex: Functional studies in ALS
Dr Gerald Pfeffer, University of Calgary	Glymphatic alterations in amyotrophic lateral sclerosis
Sarah Jacob, University of Calgary	Oral microbiome analysis in ALS demonstrates differences associated with limb vs bulbar onset cases
Dr Tefani Perera, University of Calgary	Amyotrophic Lateral Sclerosis Functional Rating Scale (ALSFRS) Thresholds Predict Risk of Feeding Tube Insertion
Dr Lola Lessard, University of Ottawa	Metabolic defects in Type 1 Myotonic Dystrophy: a potential therapeutic target
Dr Lola Lessard, University of Ottawa	Contribution of major histocompatibility complex class II immunostaining in distinguishing idiopathic inflammatory myopathy subgroups: a histopathological cohort study
Tyler Soule, University of Calgary	Single nucleus RNA sequencing reveals unique myonuclei populations in late-onset myopathy

## Topics in neuromuscular disease

### Clinical diagnosis and neurophysiology

Lectures on topics in neurophysiology focused on diagnostic challenges with a clinical emphasis. Diagnostic mimics in the electromyography laboratory were presented. This included common lower and upper extremity consultation questions including reviewing literature that showed up to ∼50% of referrals for carpal tunnel syndrome had alternative clear musculoskeletal diagnosis.^1^ Clinical pearls for diagnosing and differentiating clinical presentations such as scapular winging, thoracic outlet syndrome and common causes of musculoskeletal pain were discussed.

A subsequent lecture provided case-based teaching on the autonomic testing battery and quantitative sensory testing. This included post-COVID dysautonomia and its high prevalence affecting more than one third of patients with very heterogenous presentations,^2^ cases of small fiber neuropathy including acute small fiber neuropathy^3^ along with other causes.

A practical overview of the challenges of performing EMG in ICU settings was provided. Key barriers include electrical noise, patient sedation, and equipment limitations, all of which can increase the risk of misinterpretation. Differential diagnosis can be broad and overlapping, including common ICU-acquired conditions such as critical illness polyneuropathy and myopathy, and less frequent causes such as Guillain-Barré syndrome, botulism, and inherited neuromuscular disorders. ICU-acquired weakness affects up to 50 percent of patients after seven days, with mixed neuropathic and myopathic features predominating. Risk factors include prolonged mechanical ventilation, sepsis, renal failure, and exposure to agents such as corticosteroids and propofol. Ventilator-induced diaphragm dysfunction was mentioned as an underrecognized but significant contributor to weaning failure. ICU EMG requires avoidance of overinterpretation and focusing on reliable findings such as conduction block or temporal dispersion.

### Patient-reported outcomes

The importance of patient-reported outcomes was emphasized in two lectures. Experience from the UBC Neuromuscular Clinic with subcutaneous immunoglobulin therapy (SCIG) in comparison to IV delivery (IVIG) highlighted value-based health care as a framework for patient centered care.^4^ This topical area also recently had reports from other groups. Incorporation of patient reported outcome measures (PROMs) and functional measures are useful in improving clinical care, but also an important part of clinical trial design.^5^ Preliminary cost-effectiveness analyses were also discussed.

Spinal muscular atrophy was separately discussed as a condition in which patient reported outcomes are increasingly used, especially for bulbar function. There was a national Canadian consensus effort to standardize a core toolkit of outcome measures for adults with SMA.^6^ This was developed through a modified Delphi process, including motor, respiratory, and functional assessments. Identified gaps include limited use of patient-reported outcome measures and the absence of validated tools for assessing bulbar function, which includes speech, swallowing, and chewing. Drawing on patient perspectives, bulbar function is a high priority for people living with SMA, often considered more important than mobility in daily life.^7,8^ Despite this, existing tools rarely capture improvements in these areas, even when treatment leads to meaningful gains.^9^ Patient reported outcome tools are currently being piloted in Canadian clinics, such as the Eating Assessment Tool (EAT-10) and the Voice Handicap Index.^10^ Following this experience, the revised national toolkit now uses a continuum-based approach rather than fixed functional categories, allowing clinicians to select the most appropriate measure based on individual needs.^6^ The updated framework includes new patient-reported outcome measures and preliminary bulbar function assessments, while recognizing the need for SMA-specific tools in this area.

### Updates in therapy for neuromuscular disease

The treatment update on myasthenia gravis reviewed the role of thymectomy for non-thymomatous myasthenia gravis demonstrating that patients that received thymectomy had a higher probability of achieving remission or minimal manifestation status compared to control.^11^ More recent study results suggested thymectomy was associated with increased all cause mortality, risk of cancer and risk of autoimmune disease, but this remains controversial and further studies are underway.^12^ Level B evidence for intravenous immunoglobulin therapy (IVIg) in treating moderate to severe myasthenia gravis^13^ was reviewed along with examining studies looking at transition between intravenous to subcutaneous immunoglobulin treatment.^14^ The presentation then concluded with reviewing more recent targeted immunotherapies including complement inhibition with eculizumab,^15,16^ ravalizumab^17^ and zilucoplan.^18^ Studies targeting neonatal Fc Receptor inhibition with efgartigimod,^19^ rozanolixizumab^20^ and nipocalimab^21^ were also discussed.

Hereditary transthyretin amyloidosis (hATTR) was reviewed, discussing the wide spectrum of clinical presentations given the multisystem involvement.^22^ Presentations with polyneuropathy and high prevalence of autonomic manifestations were emphasized.^23^ Early diagnosis is critical and several red flags can suggest diagnosis,^24^ crucial given the treatment options for a once progressive fatal disease. Genetic therapies including siRNA therapy, patisiran,^25^ and antisense oligonucleotide therapy, inotersen.^26^ Additional therapies discussed, including vutrisiran^27^ and eplontersen,^28^ have subsequently been approved by Health Canada.

Spinal muscular atrophy (SMA) has shifted from being a leading cause of infant mortality to a treatable condition, thanks to the implementation of newborn screening and the availability of disease-modifying therapies.^29,30^ Data presented from Ontario and Alberta, demonstrated that treatment initiated within the first month of life, particularly for individuals with two or three *SMN2* copies, is associated with significantly improved motor outcomes, including higher rates of independent sitting and walking.^31,32^ These findings, supported by both clinical trials and real-world evidence, reinforce the importance of presymptomatic treatment.^33^ There remain complexities of managing individuals with four *SMN2* copies, where disease onset may be delayed.^34^ Accurate *SMN2* copy number determination, ideally through expert laboratories, is essential to avoid missed therapeutic windows.^35^ Rare cases, such as compound heterozygotes, may still be missed with screening and require rapid diagnostic response and personalized care planning.

## Transdisciplinary session

Muscular Dystrophy Canada (MDC) was introduced as a pan-Canadian patient organization that serves individuals living with more than 160 neuromuscular conditions, both inherited and immune-mediated. Rooted in the principle of “nothing about us without us,” MDC integrates the voices of people with lived experience across all levels of its work, from governance to service delivery. The organization operates independently of government funding, allowing it to act both as a direct service provider and a strong, credible voice in public policy. MDC actively engaged in shaping rare disease strategies, with recent success in influencing the addition of spinal muscular atrophy to newborn screening programs across Canada.^36^ Patients often experience prolonged diagnostic journeys, repeated misdiagnoses, and delayed access to specialized care.^37^ After diagnosis, individuals continue to face limited availability of multidisciplinary services, substantial out-of-pocket costs for assistive devices and therapies, and fragmented navigation across healthcare, insurance, and social systems.^37^ As individuals live longer with neuromuscular conditions due to advances in treatment, their needs evolve and intensify. MDC helps bridge these gaps by offering personalized, wraparound support. MDC's approach aligns with social prescribing and person-centered care, empowering individuals to define their goals and access the tools and supports necessary to live well. MDC also plays a central role in Canada's neuromuscular research ecosystem, facilitating clinician and patient collaboration, supporting knowledge translation, and advancing equitable access to innovation.

Rehabilitation is an evolving area in the context of gene therapies for neuromuscular disorders. Disease-modifying treatments have significantly improved survival and motor outcomes, particularly in SMA, where early intervention leads to substantial functional gains such as independent sitting and ambulation.^38^ There is a growing need to address other health domains, including cognitive development,^39^ cardiopulmonary function, skeletal integrity, and psychosocial adaptation.^40,41^ There are emerging data pointing to executive function and attention deficits in some treated individuals with SMA and Duchenne muscular dystrophy (DMD), as well as persistent cardiomyopathy in DMD, which may be attributed to limited therapeutic penetration of the brain and cardiac tissue. Cardiac outcomes in individuals with DMD can be improved with proactive heart failure management.^42^ Other goals for rehabilitation should go beyond traditional motor-focused goals and include educational attainment, workforce participation, emotional well-being, and future considerations such as reproductive health and family planning.

WCNMC hosted the first presentation of data from the BIND Study (Burden of Inherited and Inflammatory Neuromuscular Disorders in Canada), the largest national analysis to date of the economic and social impact of neuromuscular disease.^43^ The study included over 1400 respondents from across Canada, representing individuals with neuromuscular conditions and their caregivers.^44^ It revealed that only 22 percent of adults with neuromuscular disease reported paid employment, compared to 61 percent in the general Canadian population (56% after matching for age and sex). More than 80 percent reported financial hardship attributable to their condition. Participants incurred an average out-of-pocket cost of $24,000 over five years, with more than half drawing from retirement savings. Despite having private insurance, 13.0 percent of participants reported skipping medications and 12.5 percent missed medical appointments due to cost. Comparative analyses showed that the financial toxicity experienced by people with neuromuscular disorders exceeds that reported in other chronic diseases and even advanced cancer. For example, the average COST score,^45^ a validated measure of financial distress, was 17.2 for neuromuscular disease, which is lower (more severe) than reported COST scores for cancer^45,46^ and comparable to multiple sclerosis.^47^ The data also highlight significant systemic inequities, with access to genetic testing, therapies, assistive equipment, and multidisciplinary care varying widely across provinces.

## Genetic neuromuscular disease

Pompe disease (glycogen storage disorder type II) has seen transformative reductions in infant mortality due to enzyme replacement therapy with recombinant human acid alpha-glucosidase (rhGAA), but challenges remain.^48^ These include variable skeletal muscle penetration and response to rhGAA, immunologic response limiting rhGAA efficacy in children with no natural GAA production (CRIM-), and targeting skeletal muscle with genetic therapies. Novel strategies attempt to overcome these challenges, including open label studies of high dose rhGAA to enhance skeletal muscle penetration,^49^ concurrent rhGAA and immune modulation in CRIM- children,^50^ chaperone molecules to enhance myocyte penetration of rhGAA^51,52^ and centryin conjugated small interfering RNA that reduce glycogen synthesis,^53^ and ex-vivo genetic therapies that may mitigate viral vector associated immune mediated toxicity.^54^

Congenital myasthenic syndromes (CMS) were reviewed with a focus on CMS caused by pathogenic variants in *DOK7* (DOK7-CMS). Widely variable phenotypes range from severe infantile hypotonia to atrophy, scoliosis and truncal and respiratory involvement. Some forms of CMS do not respond and can even worsen with cholinesterase inhibitors, in particular DOK7- and COLQ-CMS, which can respond to beta adrenergic agonists, and slow channel CMS, which can respond to fluoxetine. Updates in this area include the recent discovery of agonist antibodies that phosphorylate muscle-specific tyrosine kinase (MuSK) and restore normal acetylcholine receptor clustering at the neuromuscular junction, normally impaired in DOK7-CMS. These antibodies restore survival in *DOK7* mutated mice,^55^ and a phase 1b clinical trial is now underway to test a novel MuSK agonist antibody in a small number of adults with DOK7-CMS.^56^

Genetic myopathies are commonly comorbid with cardiac dysfunction, which was reviewed in a session focused on cardiac management of neuromuscular patients. Advanced cardiovascular care is needed in people with dystrophinopathies^57^ as mean survival in Duchenne muscular dystrophy (DMD) has extended from late-teens in the 1960s to above 30-years currently.^57,58^ Cardiac MRI is important to characterize myocardial fibrosis before structural changes are evident on echocardiogram (ECG).^59^ Early initiation of angiotensin converting enzyme inhibitors (ACEi) by age 8–10 years followed by staged introduction of additional heart failure therapies, can have a dramatic impact on survival. In myotonic dystrophy type 1 (DM1) one challenge is predicting optimal timing of implantable pacemaker and defibrillator placement for cardiac conduction defects and preventing sudden cardiac death. Practice guidelines are largely based on identifying specific ECG abnormalities that predict increased risk of sudden cardiac death,^60^ but people with DM1 are at risk of severe cardiac conduction disease even when ECG is normal.^61^

### Genetic founder diseases

Several founder diseases have a high prevalence in different Canadian regions. The Saguenay-Lac-St-Jean (SLSJ) region has a high prevalence for numerous genetic conditions, including DM1, due to a triple founder effect.^62^ Some of the extensive variability may be related to genetic modifiers and the founder population in SLSJ is well suited for this, given the genealogy data available for large families, which correlates to haplotype information.^63^ Some modifiers for DM1 have already been identified,^64^ one of which has been identified in a large SLSJ family, and is a subject of ongoing study. There is a broad pipeline of therapeutic development for DM1 and the approach to eventual therapy for this disorder will be challenged by the extensive phenotypic variability, which only loosely correlates to CTG expansion size.^65^ There are numerous important symptoms that affect patient outcomes in DM1, including muscle weakness but also including sleep disturbances,^66^ respiratory function,^67^ cognitive dysfunction,^68^ among others. DM1 also has important implications for women's health which may be under-recognized.^69,70^ Discussion of biomarkers and therapeutic strategies including exercise was provided.^71^

In Western Canada, numerous founder populations are present, among these important neuromuscular conditions prevalent in Hutterite populations of Alberta and Saskatchewan.^72^ Most relevant to neuromuscular specialists is the high prevalence of limb girdle muscular dystrophy,^73^ which has been linked to pathogenic founder variants in *TRIM32*^74^ and *FKRP*.^75^ The carrier frequency is sufficiently high that patients with both conditions have been described in these communities.^76^ Other less common conditions with neuromuscular manifestations include recessive laminopathy due to *LMNA*,^77^ as well as disorders associated with *TRAPPC11* and *MYH2*. Spinal muscular atrophy is also common in this population, and with their founder haplotype, always presents with milder phenotype, and patients generally remain ambulant.^78^ The carrier frequency for the various founder variants has been estimated.^79^ Neuromuscular conditions due to single gene disorders are much less common in Mennonite populations but a founder frameshift variant in *TRIP4* results in prenatal spinal muscular atrophy and congenital bone fractures.^80^ A database exists for genetic disorders in these populations for reference.^81^

Spinal bulbar muscular atrophy (SBMA, or Kennedy disease), was recently discovered to have a founder effect in Cree First Nations and Métis people of the prairie provinces. Based on initial estimates, the prevalence of SBMA is much higher in Cree First Nations, and may be 100-fold higher in people of Saulteaux ancestry.^82^ This is further supported by a high number of incident cases identified in a survey of molecular diagnostic lab testing results in Alberta, Saskatchewan and Northwest Territories.^83^ These findings are likely to be underestimated, and indeed SBMA is likely to be underdiagnosed in all populations, based on analyses from genomic databases.^84^ Further work is in progress, including an extension of the previous project, and photovoice^85^ will be used to obtain qualitative information about Indigenous experiences with SBMA.

## Research forum

The inaugural WCNMC research symposium included three presentations by researchers followed by five trainee best abstract presentations. An overview of translational research in neuromuscular disease presented an introduction to antisense oligonucleotide (ASO) therapy, including general approaches to knock down genes (steric blocking and gapmers^86^) or to increase gene function (splice switching^87^) and a review of the history of these methods to treat genetic diseases. Here, the discussion was focused to Duchenne muscular dystrophy, a severe and fatal muscular dystrophy caused by hemizygous mutations in DMD.^88^ In DMD caused by deletion of exon 52, the approved DMD therapy viltolarsen restores the reading frame of DMD by causing skipping of exon 53.^89^ Recent research using DG9 peptide facilitates entry of ASO into cardiac tissue,^90^ which is of clinical importance given the morbidity and mortality caused by cardiomyopathy in DMD. Additional challenges and future considerations for exon-skipping therapies were discussed, including the development of personalized therapies (n of 1)^91^ and design challenges (somewhat improved with the availability of novel tools,^92,93^).

Further translational work was presented on amyotrophic lateral sclerosis (ALS) as a disease influenced by genetics, sexual dimorphism, and environmental factors. Recent studies have demonstrated the importance of the gut microbiome and ALS pathogenesis using animal models.^94,95^ ALS is well known to have male predominance, with higher male incidence, earlier age of onset, faster progression, and a predilection for spinal-onset disease.^96^ This dimorphism is not understood, but data presented suggest that the microbiome may be a contributor. Using TDP43^A315T^ mice, analysis of mice by sex shows relatively longer survival for females than males.^97^ Depletion of the microbiome using antibiotics results in reduced survival and a reduction in the difference between males and females. When grown in germ-free conditions, transgenic mice have reduced survival and sexual dimorphism is no longer present. The gut microbiome may be protective against TDP43 toxicity in mice of both sexes and contribute to the sexual dimorphism of ALS. Analysis of the microbiome in these mice shows a relative enrichment of energy producing and anti-inflammatory bacteria in female animals.^97^ Future research should include a study of fecal microbiota transplantation,^98^ and probiotic supplementation.^99^

The subsequent presentation focused on glymphatic alterations in amyotrophic lateral sclerosis. Recently, a publication showed that DTI-ALPS is reduced in ALS compared to controls in a cohort of early onset ALS participants.^100^ A longitudinal analysis of Calgary participants in CALSNIC^101^ included ALS participants, controls, and some with a different motor neuron disease, primary lateral sclerosis (PLS).^102^ DTI-ALPS index was reduced in ALS participants compared to controls, but was not reduced in PLS participants.^103^ The alterations of DTI-ALPS were persistent across longitudinal measurements. An analysis of white matter lesion burden did not show any significant correlation of white matter injury to DTI-ALPS index. Differences were not observable based on sex, disease severity, disease phenotype, or progression rate, but this may have been limited by small sample size.

Five best abstract trainee presentations followed, some including recently published works.^104,105^ The winning trainee presentation was delivered by Dr Tefani Perera (University of Calgary) for her presentation, “Amyotrophic lateral sclerosis functional rating scale (ALSFRS) thresholds predict risk of feeding tube insertion”. A total of 11 abstracts were presented as posters at the conference and amongst these the winning poster was presented by Dr Lola Lessard on the topic of “Contribution of major histocompatibility complex class II immunostaining in distinguishing idiopathic inflammatory myopathy subgroups”.^104^

## Conclusion

The 2024 Western Canadian Neuromuscular Conference convened participants from across the country, underscoring the national significance of neuromuscular research, care, and lived experience. The conference emphasized the critical role of interdisciplinary collaboration, the growing importance of real-world evidence, and the need to translate emerging science into clinical practice. The conference organizers plan to host WCNMC on a bi-annual schedule, alternating with the bi-annual schedule of the Ottawa International Conference on Neuromuscular Disease & Biology.^106^ The next iteration of the WCNMC, to be hosted in Calgary from September 25–27, 2026, aims to further strengthen capacity, foster cross-provincial connections, and encourage collaboration.
